# Variability and conservation in hepatitis B virus core protein

**DOI:** 10.1186/1471-2180-5-33

**Published:** 2005-05-27

**Authors:** Benjamin M Chain, Richard Myers

**Affiliations:** 1 Department of Immunology and Molecular Pathology, University College London, 46 Cleveland St., London, W1T 4JF UK; 2 Department of Infection, University College London, 46 Cleveland St, London, W1T 4JF, UK

## Abstract

**Background:**

Hepatitis B core protein (HBVc) has been extensively studied from both a structural and immunological point of view, but the evolutionary forces driving sequence variation within core are incompletely understood.

**Results:**

In this study, the observed variation in HBVc protein sequence has been examined in a collection of a large number of HBVc protein sequences from public sequence repositories. An alignment of several hundred sequences was carried out, and used to analyse the distribution of polymorphisms along the HBVc. Polymorphisms were found at 44 out of 185 amino acid positions analysed and were clustered predominantly in those parts of HBVc forming the outer surface and spike on intact capsid. The relationship between HBVc diversity and HBV genotype was examined. The position of variable amino acids along the sequence was examined in terms of the structural constraints of capsid and envelope assembly, and also in terms of immunological recognition by T and B cells.

**Conclusion:**

Over three quarters of amino acids within the HBVc sequence are non-polymorphic, and variation is focused to a few amino acids. Phylogenetic analysis suggests that core protein specific forces constrain its diversity within the context of overall HBV genome evolution. As a consequence, core protein is not a reliable predictor of virus genotype. The structural requirements of capsid assembly are likely to play a major role in limiting diversity. The phylogenetic analysis further suggests that immunological selection does not play a major role in driving HBVc diversity.

## Background

The evolutionary pressures that have driven Hepatitis B virus (HBV) variation remain incompletely understood. Using whole HBV genotype sequencing, this variability can usefully be classified into at least eight families (genotypes) with a characteristic geographic distribution (reviewed in [1]). Alternatively, HBV strains can be classified serologically on the basis of antibody to surface antigen (subtypes). These two classifications broadly correlate, although some subtypes appear in more than one genotype. The extent of genetic diversity reflects the evolutionary history of the virus and the rate of genomic mutation, as well as gene specific selection forces. Several models of HBV evolution have been proposed (reviewed in [2,3]) but fundamental parameters, such as the rate of interspecies transmission or the rate of nucleotide mutation (the molecular clock) remain unresolved [3]. Nevertheless, it is generally assumed that the emergence of HBV families may reflect adaptation to the genotype of the prevalent human host population [4].

The clinical course of HBV infection is very variable. Acute infections in adults are usually effectively controlled, but occasionally lead to fulminant hepatitis and death. In a proportion of individuals however, infection leads to chronic viral replication, which can lead to severe liver damage or hepatocellular carcinoma. Host factors including immune status clearly play a major role in determining clinical outcome. For example, perinatal transmission leads to up to 90% chronic carriership, while the figure is less than 10% for adults. However, pathogenicity has also been linked to virus genotype and several different mechanisms have been proposed for this observation [5,6]. Sequence changes occurring during the course of infection (longitudinal diversity) have also been extensively documented. One common example is the introduction of a stop codon in the precore region which results in downregulation of secretion of a soluble form of HBV core protein (HBVe) whose function remains unclear [7,8]. Interestingly, the downregulation of HBVe secretion is often associated with the appearance of anti-HBVe antibodies in serum, suggesting the protein itself may induce some form of immunological tolerance [7,9].

The role of adaptive immunity both in determining the course of HBV infection and in driving HBV evolution is of special interest. Although pre-existing antibody to HBV surface protein (HBVs) (for example in vaccinated individuals) clearly provides strong protection, antibody to this antigen in natural infection is a late event, usually subsequent to effective control for viremia. In contrast, antibody to HBV core (HBVc), although this protein is internal to the virion, occurs early in infection in almost all infected individuals, irrespective of their ability to control viral replication [10]. T helper and cytotoxic responses to several proteins of HBV have also been detected, and the presence of a higher frequency of HBV specific CTL in liver is associated with the ability to control viremia [11]. As might be predicted CTL responses are not limited to structural proteins, but recognise several non-structural viral proteins. Virus specific T cell immune responses are most readily detected in individuals who effectively control viral replication. In chronically infected individuals, these responses are often much more difficult to detect, suggesting that the chronic state is associated with the establishment of some form of immunological tolerance [12].

HBVc antigen is a small protein, whose three-dimensional structure has been determined by X-ray crystallography [13], and whose immunogenicity in terms of both antibody and T cell responses has been studied rather extensively in both mouse and man. It thus represents an ideal starting point for studies aimed at relating antigen structure to immune response. Indeed longitudinal studies on small groups of HBV infected individuals have suggested that variation is more common in B cell and T helper cell epitopes, suggesting a possible immune driven escape mechanism [14]. As a basis for further functional exploration we have documented HBVc variation in detail. In this study we have collected several hundred protein sequences of HBVc from public databases, and have re-examined variation in relation to structure, immunogenicity and genotype.

## Results and discussion

**Figure 1:**
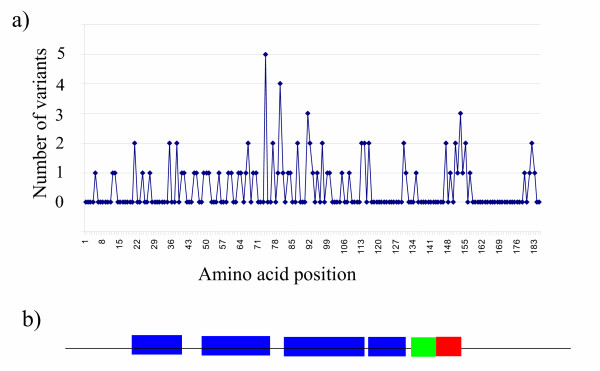
**Sequence variation along the HBVc sequence. a) **The number of polymorphisms (variants occurring in more than 1 % sequences examined) found at each position of the HBVc sequence. See additional file 1 for full data. **b) **Major features of secondary structure along HBVc [13]. Blue : α-helix Green : Proline rich loop; Red : C-terminal arm.

742 protein HBVc sequences were retrieved from the NCBI protein database after some manual curation of very short sequence fragments. The mature HBVc protein sequences 1–185 (not including the precore region) were aligned and the number of polymorphisms found at each position (defined as amino acids occurring in more than 1% of the sequences at each site) was determined (see Additional file 1). A plot of the number of variants at each position is shown in fig 1a and the major structural features, including the four alpha helices, the proline rich loop and the C-terminal arm [13] of the protein are shown in fig 1b. Over three quarters (141/185) of positions are completely invariant. Variation clustered within certain regions of the protein, with 18 of the 45 variable amino acids found within positions 59-100.

**Figure 2:**
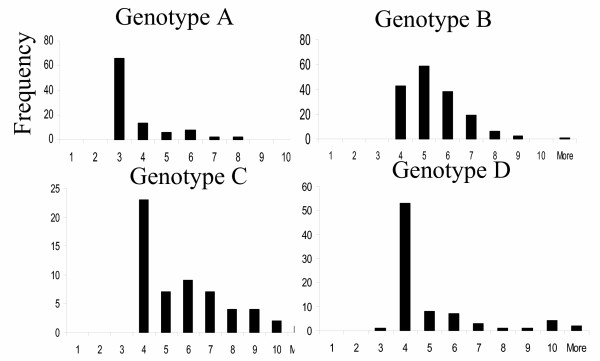
**Relationship between HBVc sequence and genotype. **The 403 sequences from viruses of known genotype were aligned and compared to the consensus. The distance of each sequence from the consensus was calculated using maximum likely-hood model (Jones-Taylor-Thornton). The frequency distribution of distance from consensus was then plotted for each genotype A-D.

Sequence variation in core protein may reflect the overall genotypic variation among HBV strains driven by drift or other unknown factors ("hitchhiking"). Alternatively, specific selection pressures may operate on HBVc driving diversity independently. In order to approach this question, the relationship between HBVc variation and HBV genotype was explored. Using text querying of the data base, a subset of 402 sequences were selected which had been assigned genotype A-D (these were the most frequent assigned genotypes within this set) by analysis of whole genome sequence or sequences outside the core region. These 402 core protein amino acid sequences were aligned and compared to each other and to the overall consensus sequence using Protdist, a Phylip program using the Jones-Taylor-Thornton model [15]. The distribution of distances between each sequence and the consensus was then plotted for each viral genotype as given in the database record (fig 2). The distance distribution profiles of the different genotypes were largely overlapping, suggesting that there was no correlation between distance from consensus and genotype.

**Figure 3:**
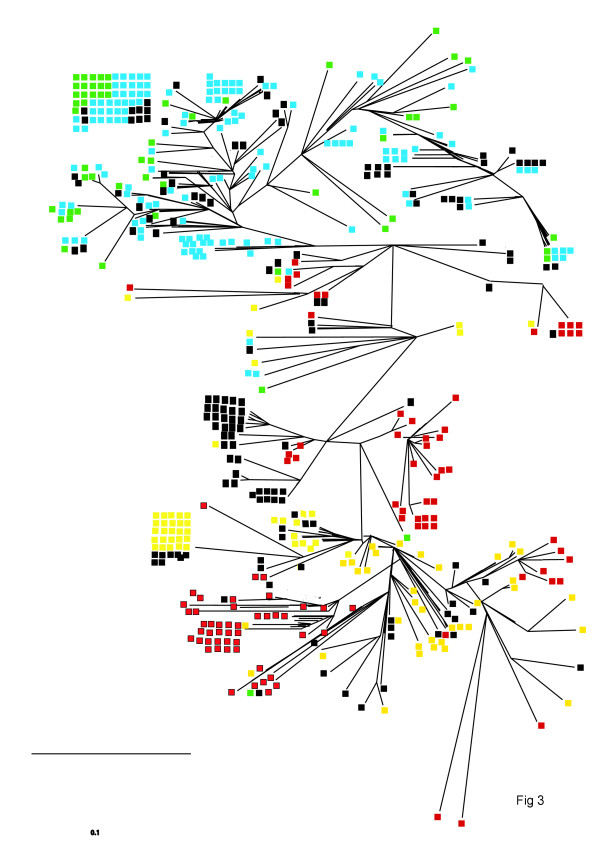
**Analysis of the relationships between HBVc sequences. **All the sequences were classified and plotted as an unrooted tree using the Fitsch Margoliash criterion (Kitsch, see Materials and Methods). Where known the genotype is indicated by colour coding (Yellow = A, Green = B, Blue = C, Red = D). Sequences of unknown genotype are in black.

In an alternative approach, the relationship between all the different core sequences was determined using Fitch-Margoliash least squares analysis, and plotted as an unrooted tree (fig 3). The different genotypes of each virus are colour coded. Although some broad clustering of genotypes was evident, a significant amount of "mixing" can be observed. Similar qualitative results were obtained when phylogeny was determined by Neighbour Joining analysis (using Phylip program Neighbor) or by parsimony (using Phylip program ProtPars).

**Figure 4:**
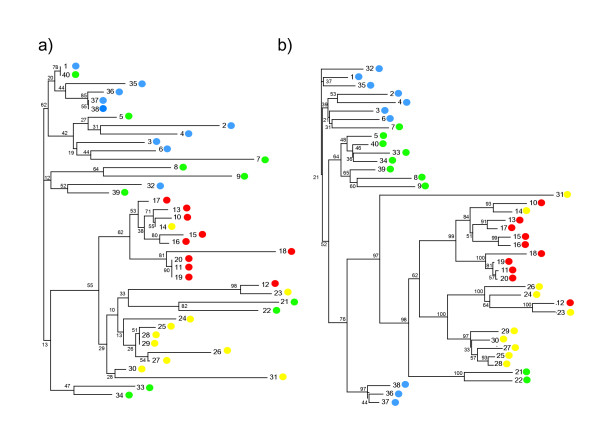
**Analysis of 40 protein and DNA HBVc sequences. **The 40 sequences detailed in Table 1 were analysed using Neighbor Joining analysis of both protein (panel a) and DNA (panel b) and plotted using TreeView. The bootstrap values (as percentage) are shown in smaller font. The accession number corresponding to each numbered sequence is shown in Table 1. The genotype is indicated by colour coding (Yellow = A, Green = B, Blue = C, Red = D).

This phenomenon was analysed in more detail in a subset of 40 full length core protein sequences selected from various sections of the tree illustrated in fig 3 (see Table 1). The phylogenetic relationship between these forty sequences was analysed again, using Neighbour-Joining (a less computation intense method) incorporating a 1000 fold bootstrap replicate in order to validate the tree topology. The results of this analysis are shown in fig 4a. In this smaller subset, genotypes A and D are reasonably well resolved, but genotypes B and C are extensively jumbled. One instance of this is illustrated by two identical sequences (1 and 40 in fig 4) derived from genotypes B and C respectively. The DNA sequences corresponding to each of these forty protein sequences were then obtained from GenBank, and analysed using the same bootstrapped Neighbour-Joining procedure. The tree obtained is shown in fig 4b. As expected, the bootstrap values are in general higher for the DNA tree (since three times as much sequence information is being analysed). The DNA sequences are somewhat more efficient at classifying B and C as separate grouping. In particular the two identical protein sequences (1 and 40 in figure) are well separated and correctly classified by the DNA phylogeny. Overall, however, significant misclassification remains, reflecting either incorrect genotyping, recombination between viral strains [16,17], or simply insufficient discrimination based on these relatively short viral sequences.

Taken together, this data suggested the forces driving the evolution of core were partially independent of the evolutionary forces driving diversification of overall genotype.

**Figure 5:**
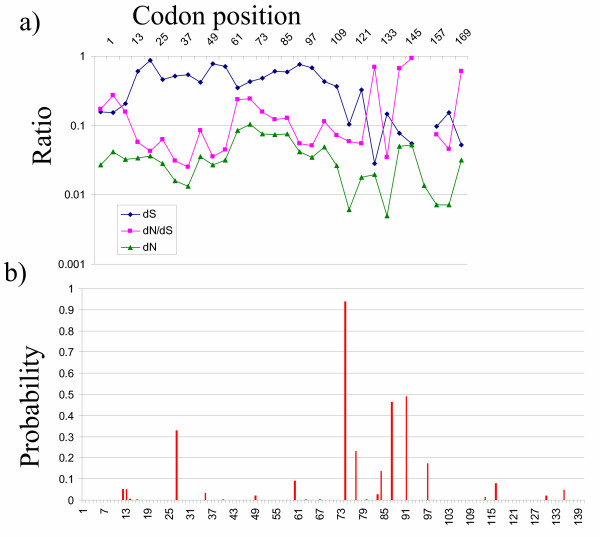
**Synonymous/nonsynonymous mutation rates and selection in HBVc. **Panel a shows the distribution of synonymous (dS) and non-synonymous (dN) substitutions (the number of such substations per site), and the ratio dN/dS along the sequence of HBVc. Gaps indicate dS = 0. Panel b shows the posterior probability p^+ ^that dN/dS>1 for each position of HBVc, as calculated by PAML. The probability that dN/dS<1 (purifying selection) is not plotted for clarity but is given by (1- p^+^).

The evolutionary pressures on sequence diversity were analysed in more detail by analysing synonymous (S) /nonsynonymous (N) nucleotide variation within the 40 viral sequences shown in Table 1. The overall distribution pattern of synonymous /nonsynonymous mutation rates is shown in fig 5a. The protein shows evidence of strong purifying selection throughout the sequence, with dN/dS ratios (using a sliding window of 36 base pairs) mostly below 0.1, The apparent dS rate was not homogenous along the length of the gene. The sharp decrease in substitution rate from around position 110 most likely reflects the start of the overlapping open reading frame of the polymerase, and this area was therefore excluded from further analysis. The identification of site-specific positive or neutral selection operating within an area of overall purifying selection has received considerable attention [18-21], and a number of methodologies have been adopted. We applied maximum-likelyhood analysis as implemented using Bayes Empirical Bayes method in the software package PAML [22] and compared a number of models of site-specific selection distribution [18]. The best likelihood value was obtained using model 8 (see Methods) which includes a positive selection subset. The posterior probabilities for the positive (ω = 1.38) classes are plotted in fig 5b. Interestingly, only one position showed a probability of >90 of being positively selected, with two more approaching 50% probability. In contrast 133 out of 140 codons showed a probability of >90% of negative purifying selection. Thus there appears to be only very limited amount of positive selection within the HBV core sequence.

**Figure 6:**
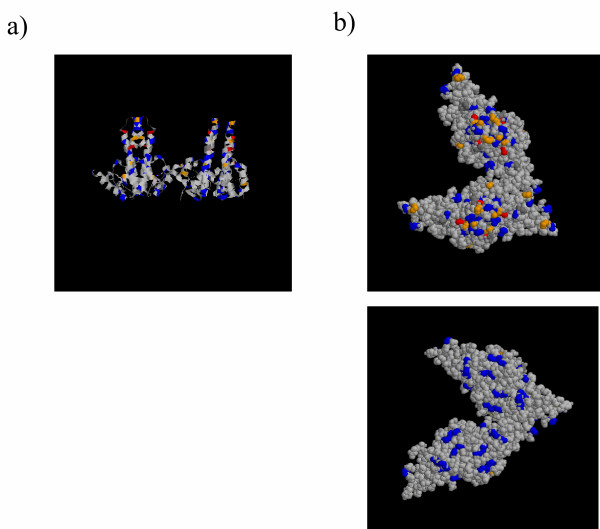
**Sequence variation in the context of capsid structure. **The crystal structure formed by four HBVc subunits was displayed and coloured using RasMol software. Positions containing 2 polymorphisms are coloured blue, 3 polymorphisms are coloured orange, and 4 or above are coloured red. Grey amino acids are invariant. a) The structure is displayed in ribbon form, showing a vertical section though the capsid, with two spikes projecting upwards, and the internal face of the capsid shown at the bottom of the picture. b) The structure is displayed in space fill. Upper Panel The structure is displayed so that the spikes and outward surface of the capsid are shown towards the viewer, and only the outer surface of the capsid is visible. Lower Panel The structure is rotated by 180 degrees so only the lower (inner) face of the capsid is visible.

The sequence information in Additional file 1/fig 1 was rationalised on the three dimensional structure of the capsid obtained from the Brookhaven Data Base. As evident in fig 6 the variation in sequence is concentrated on the spikes at the outer surface of the capsids. All the amino acids with three or more variants are found on the outer surface of the capsid (fig 6b upper panel) while the internal surface facing the capsid lumen are relatively conserved (fig 6b, lower panel).

**Figure 7:**
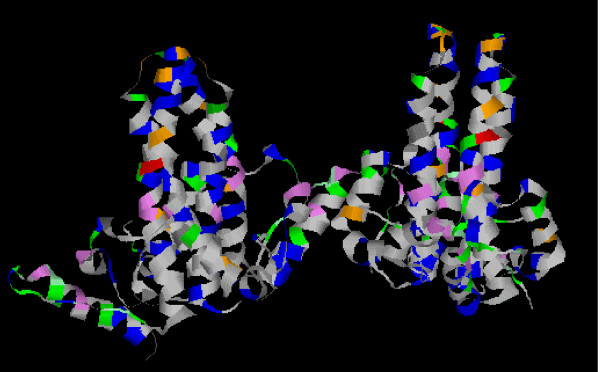
**Structural constraints and HBV diversity. **The crystal structure formed by four HBVc subunits was displayed and coloured using RasMol software. The position of the amino acid identified as essential for virion formation (violet) or for both capsid and virion assembly (green) [23] are shown. The rest of the colour coding is as shown in fig 6.

A detailed alanine mutagenesis study has been carried out mapping those amino acids critical to proper capsid formation, and/or required for envelopment and virion formation [23]. Mutation of 24 amino acids was found to block capsid formation, virion formation or both. The position of these mutations on the 3D structure is shown in fig 7. The position of this set of mutations is quite widely distributed over the structure, suggesting multiple essential interactions are absolutely required either for proper capsid assembly, or for envelope and virion assembly. However, interestingly, all but one of these 24 amino acids were found to be invariant in the data set analysed in this study. The only exception observed was at position 129, where changing proline to alanine was found to block both capsid and virion formation. Both glutamine and threonine are found in a proportion of virus sequences at this position, and further mutagenesis will be required to clarify the constraints imposed by the requirements of virion formation on the sequence at this particular position. Thus the structural requirements of virion assembly seem to impose a significant restraint on HBVc diversity. However, several positions which were found to tolerate alanine mutagenesis in terms of capsid/virion assembly were nevertheless invariant in the set of sequences examined here. The overall high degree of conservation in HBVc therefore probably reflects the multiple functions required from this protein, including control of intracellular targeting [5], pregenome/DNA polymerase packaging, capsid disassembly and viral maturation.

**Figure 8:**
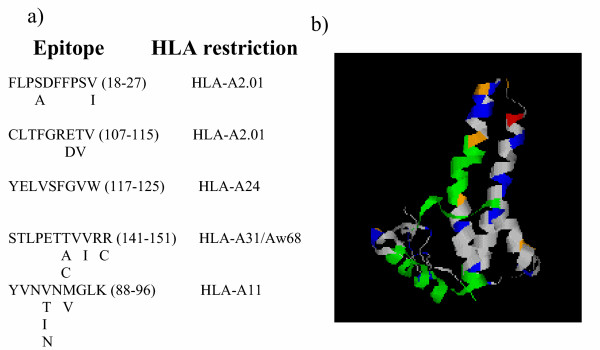
**Variation within CD8 T cell epitopes of HBVc. **Five of the best defined class I T cell epitopes from HBVc (for references see text) are shown together with alternative amino acids found at each position. The position of the epitopes (green) within the HBVc three dimensional structure is shown in the right hand panel. The rest of the colour coding is as shown in fig 6.

Pressure by the host immune system is one obvious candidate driving variation in the protein sequence of HBVc. CD8 cytotoxic cells are believed to play a key role in controlling virus replication during HBV infection [24,25]. Several CD8 T cell epitopes have been characterised in detail (e.g. [26-28]) by the use of T cell clones and lines or by elution from HLA [29]. Some of the sequences are shown in fig 7, together with the known variant sequences in the dataset analysed in this study. Although several of the epitopes lie within the more conserved inner region of the capsid, two of the epitopes show substantial polymorphism (fig 8). Interestingly, a previous paper did not find any evidence for emergence of mutations within the major CD8 epitopes during chronic HBV infection [30].

**Figure 9:**
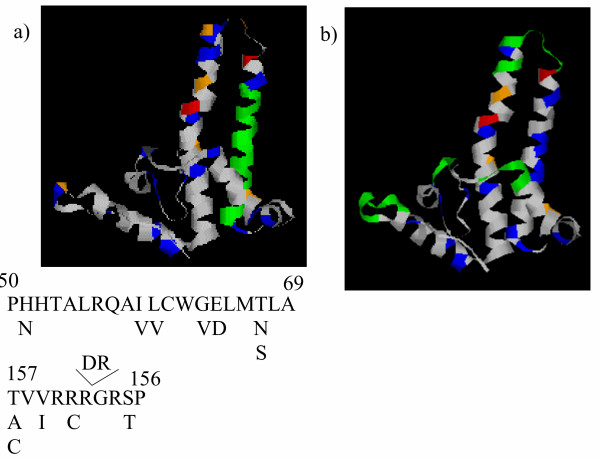
**Variation with CD4 and antibody epitopes of HBVc. **a) Two of the best defined CD4 epitopes within HBVc (taken from [31,32]) are shown together with alternative amino acids found at each position. The position of the epitope 50#150;69 is shown in the context of the HBVc three dimensional structure above(in green). The rest of the colour coding is as shown in fig 6. Epitope 147#150;156 is not shown since this region of HBVc was excluded in the crystallographic study. b) The best defined antibody epitopes (taken from [33]) are shown (in green) in the context of the HBVc three dimensional structure above. The rest of the colour coding is as shown in fig 6.

The evidence implicating CD4 T cells in HBV control remains much less clear. Furthermore, although regions containing CD4 epitopes have been described, many of the epitopes are not very well characterised. One putative "immunodominant" CD4 T cell epitope (amino acids 50-69) does contain a number of variable amino acids (fig 9a), and indeed changes in its sequence have been related directly to changes in T cell response in vivo [31]. A second epitope (core region 147-156) identified as a major target in HLADR13 individuals has also been examined in some detail [32]. Interestingly this epitope also shows considerable variation (fig 9a) including a mutation at position 151 shown to be essential for T cell recognition. The epitope also spans the region of a two amino acid insertion which is found exclusively in viruses of genotype A. Further detailed mapping of CD4 T cell recognition sites, in relation to natural sequence variation would be seem to be an area of great interest.

Finally, considerable information has been accumulated on the interaction of antibodies to HBV capsids. As shown in fig 9b[10] the major defined antibody specificities lie on the outside of the capsid structure, particularly at the tip of the spikes and at the junctions between adjacent spikes. These regions do indeed contain the majority of the HBVc sequence variation, although the most variable amino acids themselves have not been identified as known antibody contacts [33]. However, the contribution of anti-HBVc antibody to protection remains unclear, particularly since the capsid in intact virions is presumably largely shielded from antibody by the HBV envelope.

## Conclusion

This study makes use of the large number of HBVc sequences now available in public databases to characterise sequence variation in HBVc. One limitation of such an approach is that detailed clinical information associated with infection is not available, and in particular, it is not possible to examine variability in the context of the longitudinal course of an HBV infection. This is likely to be an important factor since mutations are often found to arise late in infection, associated with a variety of clinical outcomes (e.g. [34,35]). In addition few of the available sequences have been checked for their ability to make competent infectious virus, and some sequences may therefore represent non-functional proteins. However, despite these limitations, the data available does allow some general conclusions.

Overall, HBVc contains a large proportion of invariant amino acids, and a strong over representation of synonymous versus non-synonymous mutations at almost every codon. Both features suggest the presence of strong constraining forces on sequence diversity. Virion assembly is likely to provide one major constraining force [23]. As reported previously (e.g. [14]) sequence diversity appears to be clustered, and mapped predominantly to the spike and external surface of the capsid. These positions may allow greater flexibility in terms of virion assembly.

One significant consequence of the strong purifying selection is that protein sequence is a poor predictor of genotype for this gene. DNA sequence which reflects predominantly synonymous mutations, is a better discriminator, particularly in resolving genotypes B and C. Longer sequence analysis, however, is clearly necessary to obtain reliable genotyping information.

The putative role of immune selection in driving HBV core diversity is much more unclear. Direct evidence for positive selection, at least using the analysis presented here, identifies only a single amino acid (position 74 at the tip of the viral spike) as showing evidence for positive selection of diversity. Nevertheless, it is clear that several polymorphic positions lie within T or B cell epitopes. Hence, while the overall effect of immune selection on HBV sequence diversity may be small, sequence diversity may have a significant effect on the immune response at an individual level. The data analysis given here will help inform further analysis of the HBV-specific immune response. The combination of T cell and antibody recognition studies with directed mutagenesis of HBVc should determine more precisely the relationship between structure, immunity and pathogenicity.

## Methods

Human HBVc sequences were retrieved from the NCBI protein sequence database [36], limiting the search to organism = Hepatitis B virus and searching for core protein. Additional classification into genotypes A-D was done using the text search tool to look for "genotype X". A proportion of hits were verified by manual inspection.

The initial 780 hits were aligned using the EMMA program on EMBOSS (a version of Clustal)(see[37] using the BLOSUM 62 similarity matrix. The alignment was further refined by manual inspection using the sequence editor Bioedit, and very short or badly aligned sequences removed. The frequency of amino acids at each position was determined using the EMBOSS program PROPHECY. The matrix observed was converted into polymorphism frequency by setting a cut-off of 1% frequency at each position.

A phylogenetic tree of the HBVc sequences was created using the Phylip program Kitsch [38]), which uses a Fitch-Margoliash criterion based on a distance matrix obtained using the Phylip program Protdist. The tree was displayed using Treeview [39], copied in Adobe Illustrator and color coded according to genotype. Because this method is extremely processor intensive (the best tree is analysed at each iteration) it was not possible to bootstrap. Analyses of the same data were also done using nearest neighbor analysis (using the Phylip program Neighbor) and parsimony using the EMBOSS (loc cit) program EPROTPARS. Although the fine details of the trees varied between methods the overall qualitative conclusion were the same. In order to further validate the conclusions of the phylogeny, 40 sequences (shown in Table 1) chosen manually to cover the major branches of the tree shown in fig 3 were reanalysed using the Nearest Neighbour with bootstrap option (1000 bootstraps) of ClustalW [40]. The consensus tree set was plotted in Treeview and coloured in Illustrator. Similar analysis was carried out on an alignment of the DNA sequences corresponding to each protein sequence.

The analysis of synonymous/nonsynonymous mutations rates was carried out initially using the program DNASP3.0 [41] (using the Nei/ Gojobori algorithm) with a 36 base pair sliding window shifted by 9 nucleotides. A more detailed analysis at individual codons was carried out using the program PAML version 3.14 (using maximum likelihood Bayes Empirical Bayes inference, as described in [20]). Analysis was carried out using a variety of selection models, but the output represented in fig 5 used model 8 [22]. The models assume that the selection pressure (measured as ω) operating at each codon can fall within a range of different classes. Model 8 assumes a distribution of negative selection values (for all of which ω <1), or a positive selection class with ω = 1.38. This model was found to give the best likelihood estimate. The program then calculates the posterior probability (p value) that each codon within a sequence falls within a particular class.

The crystal structure of HBVc was retrieved as a pdb file from the Brookhaven database, and displayed and coloured using RasMol software (version 2.7.1.1 for Windows). Figures 6 and 7 show the structure of four identical monomers, to illustrate the spikes and their interaction, while figs 8 and 9 show a single monomer for clarity.

## Abbreviations

Hepatitis B virus HBV; Hepatitis B core protein HBVc; Hepatitis B surface protein HBVs

## Authors' contributions

RM provided bioinformatics support and expertise, and carried out the phylogenetic analysis shown in fig 4. BMC initiated the project and was responsible for the text, and the data and analysis shown in the other figures.

## Supplementary Material

Additional File 1 Amino acid polymorphisms along the HBVc sequence. Table containing all the polymorphic amino acid residues found at each position along the 742 HBV sequences analysed. Click here for file

## Figures and Tables

**Table 1:** Accession numbers and genotype for HBV sequences analysed in fig 4

Number	Accession number		Genotype
	Protein	DNA	

1.	AAK81691	AY040627	C
2.	AAO41301	AY167091	C
3.	AAP06648	AY206392	C
4.	AAL31832	AF324119	C
5.	AAP06544	AY206375	B
6.	AAP06549	AY206376	C
7.	AAL31776	AF323466	B
8.	AAL31802	AF324088	B
9.	AAP06642	AY206391	B
10.	AAP79869	AY233296	D
11.	AAP31176	AY269059	D
12.	AAR19326	AY230115	D
13.	BAC57440	AB090269	D
14.	AAL31837	AF324124	A
15.	AAF24670	AF121240	D
16.	BAD02318	AB126581	D
17.	AAL31826	AF324112	D
18.	AAP31178	AY269061	D
19.	BAA85376	AB033559	D
20.	AAP31169	AY269052	D
21.	BAC92696	AB106885	B
22.	BAC92693	AB106884	B
23.	AAR19322	AY230111	A
24.	AAL31839	AF324126	A
25.	AAP79778	AY233283	A
26.	AAL31785	AF324071	A
27.	AAP79724	AY233275	A
28.	AAP79715	AY233274	A
29.	AAP79729	AY233276	A
30.	AAP79736	AY233277	A
31.	AAL31856	AF324143	A
32.	AAG49707	AF223961	C
33.	AAP06636	AY206390	B
34.	AAF24738	AF121250	B
35.	BAA32843	AB014363	C
36.	AAP31199	AY269084	C
37.	AAL31815	AF324101	C
38.	AAL31819	AF324105	C
39.	AAL31781	AF324066	B
40.	AAF24705	AF121245	B
